# Association between Dietary Habits and Physical Function in Brazilian and Italian Older Women

**DOI:** 10.3390/nu12061635

**Published:** 2020-06-01

**Authors:** Hélio J. Coelho-Junior, Riccardo Calvani, Anna Picca, Ivan O. Gonçalves, Francesco Landi, Roberto Bernabei, Matteo Cesari, Marco C. Uchida, Emanuele Marzetti

**Affiliations:** 1 Università Cattolica del Sacro Cuore, 00168 Rome, Italy; francesco.landi@unicatt.it (F.L.); emarzetti@live.com (E.M.); 2 Applied Kinesiology Laboratory–LCA, School of Physical Education, University of Campinas, Campinas 13083-970, Brazil; uchida@unicamp.br; 3 Mãe Mariana Home, Rehabilitation Unit, Poá 08562-460, Brazil; 4 Fondazione Policlinico Universitario “Agostino Gemelli” IRCCS, 00168 Rome, Italy; riccardo.calvani@guest.policlinicogemelli.it (R.C.); anna.picca@guest.policlinicogemelli.it (A.P.); 5 Center of Health Sciences, University of Mogi das Cruzes, Mogi das Cruzes 08780-911, Brazil; ivanogedfisica@gmail.com; 6 Department of Clinical Sciences and Community Health, Università di Milano, 20122 Milan, Italy; matteo.cesari@unimi.it; 7 Geriatric Unit, Fondazione IRCCS Ca’ Granda Ospedale Maggiore Policlinico, 20122 Milan, Italy

**Keywords:** physical performance, sarcopenia, frailty, nutrition, protein intake

## Abstract

The present study investigated and compared the patterns of dietary protein intake and physical function in Brazilian and Italian older women. Seventy-five Brazilian older women were recruited in a community senior center. Fifty-three age-matched Italian older women were selected from participants of the Longevity check-up (Lookup) 7+ study. In both samples, physical performance was evaluated by isometric handgrip strength (IHG) and five-time sit-to-stand (5 × STS) tests, while diet was assessed through 24-h recall. Results indicated that Brazilian women had a higher intake of plant-based protein (52.7% vs. 30.5% kcal), while Italian women consumed greater amounts of animal-derived protein (29.7% vs. 41.5% kcal). In Brazilian women, the binary logistic regression analysis indicated that body weight-adjusted protein consumption was associated with IHG adjusted by body mass index and with 5 × STS performance. In the Italian sample, the intake of isoleucine, leucine, and valine was significantly associated with 5 × STS performance. Our findings indicate that Brazilian and Italian community-dwelling older women show different patterns of protein intake, with higher consumption of plant-based protein in the Brazilian sample and higher ingestion of animal-derived protein in the Italian subgroup. These dietary patterns may differentially impact the relationship between physical function and protein intake observed in Brazilian and Italian older women.

## 1. Introduction

Physical function involves the ability to integrate and convert physiological stimuli into a muscular action (e.g., walk) that allows interaction between the person and the environment. Dramatic changes are observed in physical function over the course of life, such that motor abilities are significantly improved during childhood and adolescence [1], whereas they progressively decline past the age of 30 years [2,3]. Such a decrease is more pronounced in women, likely due to hormonal changes in menopause [2,4].

Age-related physical dysfunction is closely associated with a wide range of negative health-related outcomes, such as falls, cognitive impairment, loss of independence, disability, nursing-home placement, and death [5,6,7,8,9]. This evidence instigated the World Health Organization to indicate the maintenance of physical function as a central element to foster healthy aging [10].

Nutrition is argued as one of the main strategies to counteract the loss of physical function with aging [11,12]. In particular, adequate protein intake may help maintain muscle mass and possibly physical function through fueling muscle protein synthesis [13,14]. Several studies have investigated the relationship between the quantity and quality of dietary protein and physical function in older adults [15,16,17,18,19]. This knowledge allowed for the generation of nutritional recommendations for older adults [20,21,22]. However, such recommendations only provide general directions, without considering cultural and regional factors [23,24,25] that may impact adherence to nutritional advices [26]. Hence, guidelines accounting for region-specific dietary habits are needed to optimize nutrition in older adults.

As an initial attempt to fill this gap in knowledge, the present study was undertaken to determine possible differences in dietary protein intake and physical function between Brazilian and Italian older women living in the community. We also explored whether physical function was associated with specific protein ingestion patterns in the two participant subsets.

## 2. Materials and Methods

The study was approved by the Research Ethics Committee of the Universidade de Mogi das Cruzes (Mogi das Cruzes, Brazil; protocol no: 621–614) and by the Università Cattolica del Sacro Cuore Ethics Committee (Rome, Italy; protocol no: A.1220/CE/2011). All study procedures were conducted in compliance with the Declaration of Helsinki and the Resolution 196/96 of the National Health Council. All participants were thoroughly informed about the study procedures before providing written consent.

### 2.1. Study Participants

#### 2.1.1. Brazilian Study Sample

Brazilian older women were recruited by convenience from the Community Senior Center of City of Poá “Cantinho do Idoso” (CCI) located in Poá, Brazil. The CCI is a major initiative to foster physical activity in older adults in the region of Poá and is managed by the municipal government. The study was advertised through posters placed in the CCI. People were also invited to participate by direct contract. Those who accepted the invitation were requested to provide informed written consent. Afterwards, candidate participants were asked to schedule an appointment at the CCI to perform the evaluations. Poá is a city located in the southern area of São Paulo with a population of approximately 100,000 people, of whom ~3.5% are 60 years or older [27]. Nearly 2000 older adults attend the center weekly to perform flexibility, aquatic and/or multicomponent physical exercises, dance classes, adapted sports, cognitive stimulation therapy, and/or to receive nursing and medical care. There is no attendance control.

Candidate participants were considered eligible if they were 60 years or older, lived independently, possessed sufficient physical and cognitive abilities to perform all the measurements required by the protocol, and provided written informed consent. Exclusions occurred if candidates had suffered a cardiac or cerebrovascular event in the previous year or if they were on hormone replacement, nutritional therapy, or psychotropic drugs. Older women who consumed dietary supplements were also excluded.

#### 2.1.2. Italian Study Sample

Italian older women were selected from the Milan EXPO 2015 database of the Longevity check-up (Lookup) 7+ project [3,28,29,30]. The Lookup 7+ project is an ongoing initiative developed by the Department of Geriatrics of the Università Cattolica del Sacro Cuore. The project started on 1 June 2015 and was designed to promote the adoption of healthy lifestyles by raising awareness in the general population on major lifestyle behaviors and risk factors for chronic diseases [28]. A team of medical doctors, researchers, and nutritionists assess people visiting public places (e.g., malls, exhibition centers) and those adhering to prevention campaigns launched by the Department of Geriatrics of the Università Cattolica del Sacro Cuore. During Milan EXPO 2015, 3206 persons from different Italian regions were evaluated in two separate periods: 1 June 2015–15 June 2015 (Marche Region pavilion) and 1 September 2015–31 October 2015 (Casa Ferrarini pavilion).

For the present study, candidate participants were considered eligible if they were 60 years or older and provided written informed consent. Exclusions occurred if candidates were unable to perform the functional tests. To ensure that the two groups were comparable, Italian older women were matched with the Brazilian sample for mean age (within 5 years). Individuals were filtered in the database according to nationality (i.e., Italian), sex (i.e., female), and age (70.5–80.5 years).

### 2.2. Anthropometric Measurements

In both samples, analogue medical scales and standard stadiometers were used to measure body weight and height, respectively. The body mass index (BMI) was subsequently calculated as body weight (kg) divided by the square of height (m).

### 2.3. Dietary Assessment

In both samples, food intake was assessed using a 24-h recall. This method provides a quantitative and subjective estimation of food consumption during the previous 24 h. Trained researchers asked participants to recall all foods consumed on a meal-by-meal basis, including snacks, in the last day. Interviews occurred on Tuesdays, Wednesdays, Thursdays, and Fridays to avoid a possible bias associated with the weekend. Participants were requested to describe cooking methods (e.g., fried, grilled, roasted), amounts in portions, product brands, sauces, spices, and condiments consumed, and the use of dietary supplements. The amounts of beverages consumed were also recorded, and participants were asked to specify if and how beverages were sweetened. Diet composition was estimated using the NutWin software, version 1.5 (Universidade Federal de São Paulo, Brazil) for the Brazilian subset [31,32] and MètaDieta (ME.TE.DA, LLC, San Benedetto del Tronto, Italy) for the Italian sample [33].

### 2.4. Physical Function Assessment

#### 2.4.1. Isometric Handgrip Strength Test

Isometric handgrip strength (IHG) was measured using a Jamar^®^ (Sammons Preston, Bolingcobrook, IL, USA) and a North Coast hydraulic dynamometer (North Coast Medical, Inc., Morgan Hill, CA, USA) in Brazilian and Italian older women, respectively. Participants performed one familiarization trial and one measurement trial with each hand, and the result from the stronger side was used for the analyses [2,3,29].

#### 2.4.2. Five-Time Sit-To-Stand Test

In both samples, participants were thoroughly instructed on the requirement of the five-time sit-to-stand (5 × STS) test by trained assessors. Following a single-repetition demonstration by the assessor, participants were invited to stand up once from the chair with their arms folded across the chest. Then, participants were asked to stand up from the chair five times in a row as quickly as possible. A standard armless chair, 43−47 cm in height was used. The chair’s back was placed against a wall to ensure safety and stability. A digital stopwatch was started when the participant raised their buttocks off the chair and was stopped when the participant was seated at the end of the fifth stand [2,3,29,34].

### 2.5. Statistical Analysis

The normality of data was ascertained using the Kolmogorov–Smirnov test. All variables were normally distributed, except for data on animal protein intake, both absolute and relative to total kcal, in the Brazilian sample. Outlier detection was performed by visual inspection of histograms. Daily animal- and plant-based protein intake was furthest from the rest in one Brazilian and one Italian participant, respectively. However, values were still plausible and were therefore retained in the analysis. Differences in continuous variables between groups (i.e., Brazilians and Italians) were assessed by the Student’s t-test for independent samples or Mann–Whitney U test, as appropriate, while the χ^2^ test was used to investigate differences in categorical variables. The median values were chosen as the cutoffs, as following: IHG (Brazilians: 22 kg; Italians: 14 kg), IHG/BMI (Brazilians: 0.8; Italians: 0.5), 5 × STS (Brazilians: 11.3 s; Italians: 14.7 s), age (Brazilians: 66 years; Italians: 77 years), BMI (Brazilians: 28.7 kg/m^2^; Italians: 29.7 kg/m^2^), relative protein consumption (Brazilians: 23.6%; Italians: 17.7%), relative animal-based protein intake (Brazilians: 41.4%; Italians: 67.3%), relative plant-based protein intake (Brazilians: 53.7%; Italians: 29.0%), body weight-adjusted daily protein consumption (Brazilians: 1.0 g∙kg^−1^∙day^−1^; Italians: 1.0 g∙kg^−1^∙day^−1^), total protein intake (Brazilians: 68.8 g; Italians: 64.6 g), animal protein intake (Brazilians: 29.0 g; Italians: 41.8 g), plant-based protein intake (Brazilians: 34.8 g Italians: 18.3 g), isoleucine intake (Brazilians: 2232.9 mg; Italians: 2480.8 mg), leucine intake (Brazilians: 4184.6 mg; Italians: 4646.2 mg), valine intake (Brazilians: 2573.7 mg; Italians: 2943.2 mg). Independent variables with a *p* value <0.05 at the χ^2^ test were included in a univariate logistic binary analysis. To be considered as an independent variable associated with physical function, results were required to have a *p* value <0.05 and a 95% confidence interval (CI) that did not encompass the value of 1. All analyses were conducted using the IBM SPSS Statistics, version 20.0, software (IBM Corp., Armonk, NY, USA).

## 3. Results

### 3.1. Characteristics of Study Participants

Data from 128 older women, 75 Brazilians and 53 Italians, were analyzed. The main characteristics of study participants according to country are shown in Table 1. Brazilian women had better performance on the IHG (*p* < 0.001), IHG/BMI (*p* = 0.081), and 5 × STS (*p* < 0.001) tests, as well as higher total (*p* = 0.043) and relative (*p* < 0.001) daily protein intake. A differential pattern of protein intake was observed between groups, with Italian women reporting higher absolute and relative intake of animal protein (*p* < 0.001 for both). On the other hand, Brazilian women reported a greater absolute and relative consumption of plant-derived protein (*p* < 0.001 for both).

### 3.2. Pearson’s Correlation between Physical Function and Dietary Characteristics According to Country

Relationships between physical function and dietary characteristics according to country are shown in Table 2. Performance of the IHG strength (*p* = 0.04) and 5 × STS tests (*p* = 0.01) was associated with BMI in Brazilian older women. In addition, body weight-adjusted daily protein intake was associated with IHG/BMI (*p* = 0.002) and 5 × STS tests (*p* = 0.04). In Italian women, isoleucine (*p* = 0.03), leucine (*p* = 0.03), and valine (*p* = 0.02) intakes were associated with the 5 × STS test performance.

### 3.3. Binary Logistic Regression for Physical Function and Dietary Characteristics According to Country

Odds ratios and 95% CI for physical function are reported in Table 3. The binary logistic regression analysis indicated that, in Brazilian women, body weight-adjusted protein intake was associated with IHG/BMI (*p* = 0.003) and the 5 × STS test (*p* = 0.04). In Italian women, isoleucine (*p* = 0.04), leucine (*p* = 0.04), and valine (*p* = 0.02) intakes were significantly associated with the 5 × STS test.

## 4. Discussion

The present study investigated differences in physical function and dietary characteristics between Brazilian and Italian older women living in the community. Our results revealed that Brazilian older women had better physical function and higher intake of plant-based protein, while higher animal protein consumption was observed in Italian older women. Furthermore, we tested the hypothesis that physical function would be differentially associated with specific patterns of protein intake. In this regard, we observed that body weight-adjusted protein consumption was significantly associated with IHG/BMI and 5 × STS tests in the Brazilian sample. Intake of the branched-chain amino acids (BCAAs) isoleucine, leucine, valine was associated with 5 × STS performance in Italian participants.

These results provide an interesting perspective about the influence of sociocultural factors on dietary consumption and physical function. In Brazilian women, the higher intake of plant-based protein may reflect the consumption of meals rich in legumes and vegetables, as it is commonly observed in this country [35,36]. Beans, for example, are among the most frequently consumed foods in Brazil [36], were present in at least one of the main meals (i.e., lunch or dinner) in Brazilian participants, and are a major source of plant protein [37]. An additional explanation for the higher consumption of non-animal protein in Brazilian women may be linked to the fact that meat has long been attributed a great symbolic value due to its high cost, making it synonymous with high social status food [38]. This traditional connotation of meat might still have an impact on food choices in Brazil [38].

One would have expected that the dietary pattern observed in the Brazilian sample was instead more likely to be found in Italian women, because legumes and vegetables are major elements of the Mediterranean diet [39]. However, our Italian sample was mainly composed of people living in northern Italy, whereas adherence to the Mediterranean diet is higher in the south [23].

As previously mentioned, the different patterns of protein consumption may have influenced the relationship between protein intake and physical function. Indeed, in the Brazilian sample, a body weight-adjusted protein consumption ≥1.0 g∙kg^−1^∙day^−1^ was significantly associated with the performance on the IHG/BMI and 5 × STS tests.

A possible explanation for these findings may reside in the fact that plant-based protein contains smaller amounts of essential amino acids and stimulates protein synthesis as well as inhibits protein breakdown to a lesser extent than animal-based protein [40]. This observation has led to the hypothesis that greater amounts of plant-based protein may be necessary to elicit the same muscle protein anabolism evoked by smaller quantities of animal-based protein [37]. In this regard, we recently observed that plant-based protein intake was significantly associated with walking speed in Brazilian older adults who consumed two-fold more vegetable protein than the Framingham cohort [31,41].

On the other hand, physical function was significantly associated with the intake of isoleucine, leucine, and valine in Italian older women. Notably, animal protein, which made up approximately 60% of protein consumed by Italian women, is the main source of BCAAs [42]. Once absorbed, BCAAs, especially leucine, stimulate muscle protein synthesis [43] via activation of mammalian target of rapamycin [44]. BCAAs have been attributed a key role in the maintenance of muscle mass and physical function during aging [37]. Furthermore, lower systemic concentrations of BCAAs have been found in older adults with sarcopenia [44]. It may therefore be hypothesized that the association between BCAA intake and physical function in Italian older women was linked to a high intake of animal-based protein.

It is worth mentioning that the median values of body weight-adjusted protein intake in Brazilian older women were higher than the estimated average requirement (EAR, 0.66 g∙kg^−1^∙day^−1^) and recommended dietary allowance (RDA, 0.8 g∙kg^−1^∙day^−1^). This finding is in line with a recent systematic review and meta-analysis that found better physical performance in older adults whose protein intake was higher than the RDA [15]. Indeed, while the RDA of protein is sufficient to avoid protein inadequacy in inactive or low-active adults, it may not provide an adequate amount of protein to maintain functional status in older adults [20]. Noticeably, the extraction of dietary amino acids by the splanchnic bed is increased in old age, which translates into lower peripheral amino acid availability in the postabsorptive state [45]. Moreover, the aged muscle shows a state of anabolic resistance such that protein synthesis in response to protein ingestion or amino acid infusion is blunted [46]. Altogether, these findings support the need of increasing the protein RDA for older adults to maintain health and maximize function [47,48], taking into account region-specific dietary patterns and cultural factors.

Some limitations of the present study need to be acknowledged. First, different recruitment strategies were used for the two participant cohorts, such that Brazilian women were enrolled in a single city, while the Italian sample included people from different regions. The possibility cannot be ruled out that a random multicenter Brazilian sample might have produced different results. In addition, analyses were conducted without a priori power calculation in a relatively small number of participants. Body composition [18], physical activity levels [17], frailty status [16,19], and oral health [49], which are associated with protein consumption and physical performance in older adults, were not controlled for in the analyses. Moreover, the use of nutritional supplements was an exclusion criterion in the Brazilian sample, but not in Lookup 7+. It should also be mentioned that the cutoff value for body weight-adjusted protein intake was set at 1.0 g∙kg^−1^, but studies have reported that higher values may be associated with better health status and physical performance in old age [15,50]. However, the relatively small sample size did not allow us to test different cutoffs. Furthermore, the lack of repeated assessments prevented establishing measurement accuracy and precision. Finally, dietary assessment through 24h recall might have impacted the estimation of protein intake because of recollection bias and/or misinterpretation by the study staff. Hence, dietary history (e.g., food frequency questionnaire), multiple 24h recalls, or dietary records should be used in future studies to provide more accurate estimates [51].

## 5. Conclusions

Findings from the present study indicate that Brazilian and Italian community-dwelling older women show differential patterns of protein consumption, with higher intake of plant-based protein in the Brazilian sample. These patterns may have an influence on the relationship between physical function and protein intake. Indeed, body weight-adjusted protein consumption was associated with IHG/BMI and 5 × STS tests in Brazilian women. BCAA intake was instead associated with 5 × STS performance in Italian participants.

## Figures and Tables

**Table 1:** Characteristics of study participants according to country.

Variables	Brazilians (*n* = 75)	Italians (*n* = 53)
**General characteristics**		
Age (years)	75.2 ± 7.5 (60–85)	77.6 ± 5.5 (70–80)
Height (m)	1.57 ± 0.1 (1.4–1.7)	1.54 ± 0.1 (1.4–1.6)
Body weight (kg)	70.8 ± 12.8 (39–98)	70.2 ± 13.3 (44.5–96)
BMI (kg/m^2^)	28.6 ± 5.2 (16.2–38)	29.8 ± 5.6 (18.5–42.4)
**Physical function**		
IHG strength (kg)	20.0 ± 10.9 (7–42)	13.1 ± 6.8 (4–32) *
IHG/BMI	1.2 ± 3.1 (0.2–1.6)	0.4 ± 0.2 (0.1–1.2) *
5 × STS (s)	11.9 ± 3.3 (7–22)	16.7 ± 6.0 (4–14) *
**Diet**		
Total daily protein intake (g)	72.7 ± 26.8 (41–129)	63.9 ± 19.2 (26–63) *
Body weight-adjusted daily protein consumption (g kg^−1^∙day^−1^)	1.04 ± 0.41 (0.2–2.4)	1.09 ± 0.44 (0.4–1.2)
Relative daily protein consumption (% kcal)	22.9 ± 5.3 (13.9–38.3)	17.6 ± 4.7 (9–43) *
Daily animal protein intake (g)	29.7 ± 17.2 (4–71)	41.5 ± 17.7 (11–43) *
Relative daily animal protein intake (% kcal)	39.7 ± 18.5 (6.5–82.4)	63.9 ± 16.2 (17–94) *
Daily plant-based protein intake (g)	37.9 ± 17.2 (10–76)	19.0 ± 8.4 (2–29) *
Relative daily plant-based protein intake (% kcal)	52.7 ± 16.4 (6.7–89.8)	30.5 ± 13.7 (7–47) *
Daily isoleucine intake (mg)	2409 ± 1155 (835–9138)	2512 ± 921 (1119–4557)
Daily leucine intake (mg)	4437 ± 2142 (1601–9725)	4516 ± 1607 (1816–9655)
Daily valine intake (mg)	2744 ± 1314 (1066–6861)	2935 ± 1047 (1160–6508)

Data are shown as mean ± standard deviation (min-max); * *p* < 0.05 vs. Brazilians. Abbreviations: 5×STS, 5-time sit-to-stand; BMI, body mass index; IHG, isometric handgrip strength.

**Table 2:** Distribution of Brazilian and Italian older women according to physical function levels (low vs. high).

	Brazilians						Italians					
	IHG		IHG/BMI		5 × STS		IHG		IHG/BMI		5 × STS	
	Low	High	Low	High	Low	High	Low	High	Low	High	Low	High
*Age (years)*												
Low	13 (17.3%)	22 (29.3%)	17 (22.7%)	18 (24.1%)	17 (22.7%)	18 (23.9%)	12 (22.6%)	15 (28.3%)	15 (28.3%)	12 (22.6%)	13 (24.5%)	14 (26.5%)
High	22 (29.3%)	18 (24.1%)	26 (34.6%)	14 (18.6%)	20 (26.7%)	20 (26.7%)	10 (18.9%)	16 (30.2%)	14 (26.5%)	12 (22.6%)	13 (24.5%)	13 (24.5%)
*BMI (kg/m^2^)*												
Low	22 (29.3%)	16 (21.3%)	20 (26.7%)	18 (24.1%)	24 (32.0%)	14 (18.7%)	12 (22.6%)	14 (26.4%)	12 (22.6%)	14 (26.4%)	13 (24.5%)	13 (24.5%)
High	13 (17.3%)	24 (32,1%) *	23 (30.6%)	14 (18.6%)	13 (17.3%)	24 (32.0%) *	10 (18.9%)	17 (32.1%)	17 (32.1%)	10 (18.9%)	13 (24.5%)	14 (26.5%)
*Total protein intake (g∙day^−1^)*												
Low	16 (21.3%)	22 (29.3%)	24 (32.0%)	14 (18.6%)	18 (24.0%)	20 (26.7%)	14 (26.4%)	12 (22.6%)	17 (32.1%)	9 (17.0%)	10 (18.8%)	16 (30.2%)
High	19 (25.3%)	18 (24.1%)	19 (25.3%)	18 (24.1%)	19 (25.3%)	18 (24.0%)	8 (15.1%)	19 (35.9%)	12 (22.6%)	15 (28.3%)	16 (30.2%)	11 (20.8%)
*Body weight-adjusted protein intake* *(g kg^−1^∙day^−1^)*												
Low	14 (18.7%)	15 (20.0%)	23 (30.7%)	6 (8.0%)	10 (13.4%)	19 (25.3%)	8 (15.1%)	18 (34.0%)	11 (20.7%)	15 (28.3%)	15 (28.2%)	11 (20.8%)
High	21 (28.0%)	25 (33.3%)	20 (26.7%)	26 (34.6%) *	27 (36.0%)	19 (25.3%) *	14 (26.4%)	13 (24.5%)	18 (34.0%)	9 (17.0%)	11 (20.8%)	16 (30.2%)
*Relative protein intake (%)*												
Low	17 (22.6%)	21 (28.0%)	24 (32.0%)	14 (18.6%)	20 (26.6%)	18 (24.1%)	13 (24.5%)	13 (24.5%)	17 (32.1%)	9 (17.0%)	13 (24.5%)	13 (24.5%)
High	18 (24.1%)	19 (25.3%)	19 (25.3%)	18 (24.1%)	17 (22.7%)	20 (26.6%)	9 (17.0%)	18 (34.0%)	12 (22.6%)	15 (28.3%)	13 (24.5%)	14 (26.5%)
*Animal protein intake, (g∙day^−1^)*												
Low	18 (24.1%)	21 (28.0%)	22 (29.3%)	17 (22.7%)	20 (26.7%)	19 (25.3%)	12 (22.6%)	14 (26.4%)	16 (30.2%)	10 (18.9%)	10 (18.9%)	16 (30.2%)
High	17 (22.6%)	19 (25.3%)	21 (28.0%)	15 (20.0%)	17 (22.7%)	19 (25.3%)	10 (18.9%)	17 (32.1%)	13 (24.5%)	14 (26.4%)	16 (30.2%)	11 (20.7%)
*Relative animal protein intake (%)*												
Low	16 (21.3%)	22 (29.3%)	19 (25.3%)	19 (25.3%)	17 (22.7%)	21 (27.9%)	12 (22.6%)	15 (28.3%)	15 (28.3%)	12 (22.6%)	14 (26.4%)	13 (24.6%)
High	19 (25.3%)	18 (24.1%)	24 (32.1%)	13 (17.3%)	20 (26.7%)	17 (22.7%)	10 (18.9%)	16 (30.2%)	14 (26.5%)	12 (22.6%)	12 (22.6%)	14 (26.4%)
*Plant-based protein intake (g day^−1^)*												
Low	15 (20.0%)	21 (28.0%)	23 (30.7%)	13 (17.3%)	15 (20.0%)	21 (28.0%)	14 (26.4%)	14 (26.4%)	18 (34.0%)	10 (18.8%)	12 (22.6%)	16 (30.2%)
High	20 (26.7%)	19 (25.3%)	20 (26.7%)	19 (25.3%)	22 (29.3%)	17 (22.7%)	8 (15.1%)	17 (32.1%)	11 (20.8%)	14 (26.4%)	14 (26.4%)	11 (20.8%)
*Plant-based protein (%)*												
Low	20 (26.7%)	18 (24.1%)	25 (33.3%)	13 (17.3%)	18 (24.0%)	20 (26.7%)	9 (17.0%)	17 (32.1%)	13 (24.5%)	13 (24.5%)	13 (24.5%)	13 (24.5%)
High	15 (20.0%)	22 (29.3%)	18 (24.1%)	19 (25.3%)	19 (25.3%)	18 (24.0%)	13 (24.5%)	14 (26.4%)	16 (30.2%)	11 (20.8%)	13 (24.5%)	14 (26.5%)
*Isoleucine intake (mg)*												
Low	16 (21.4%)	21 (28.0%)	22 (29.3%)	15 (20.0%)	17 (22.7%)	20 (26.6%)	13 (24.5%)	13 (24.5%)	17 (32.1%)	9 (17.0%)	9 (17.0%)	17 (32.1%)
High	19 (25.3%)	19 (25.3%)	21 (28.0%)	17 (22.7%)	20 (26.6%)	18 (24.1%)	9 (17.0%)	18 (34.0%)	12 (22.6%)	15 (28.3%)	17 (32.1%)	10 (18.8%) *
*Leucine intake (mg)*												
Low	16 (21.4%)	21 (28.0%)	23 (30.7%)	14 (18.6%)	16 (21.3%)	21 (28.0%)	11 (20.8%)	15 (28.3%)	16 (30.2%)	10 (18.9%)	9 (17.0%)	17 (32.1%)
High	19 (25.3%)	19 (25.3%)	20 (26.7%)	18 (24.0%)	21 (28.0%)	17 (22.7%)	11 (20.8%)	16 (30.1%)	13 (24.5%)	14 (26.4%)	17 (32.1%)	10 (18.8%) *
*Valine (mg)*												
Low	16 (21.4%)	19 (25.3%)	22 (29.3%)	13 (17.3%)	15 (20.0%)	20 (26.6%)	13 (24.5%)	14 (26.4%)	17 (32.1%)	10 (18.9%)	9 (17.0%)	18 (33.9%)
High	19 (25.3%)	21 (28.0%)	21 (28.0%)	19 (25.4%)	22 (29.3%)	18 (24.1%)	9 (17.0%)	17 (32.1%)	12 (22.6%)	14 (26.4%)	17 (32.1%)	9 (17.0%) *

* *p* < 0.05; Abbreviations: 5 × STS, 5-time sit-to-stand; BMI, body mass index; IHG, isometric handgrip strength.

**Table 3:** Unadjusted odds ratios (ORs) and 95% confidence intervals (CIs) for physical function in Brazilian and Italian older women.

**Brazilians**				
	IHG/BMI		5 × STS	
Variable	Unadjusted OR	95% CI	Unadjusted OR	95% CI
*Body weight-adjusted protein intake*				
High	4.95	1.71–14.54	0.37	0.14–0.97 *
Low	Reference		Reference	
**Italians**				
			5×STS	
Variable			Unadjusted OR	95% CI
*Isoleucine intake*				
High			0.31	0.10–0.96 *
Low			Reference	
*Leucine intake*				
High			0.31	0.10–0.96 *
Low			Reference	
*Valine intake*				
High			0.26	0.09–0.83 *
Low			Reference	

* *p* < 0.05; Abbreviations: 5 × STS, 5-time sit-to-stand; IHG/BMI, isometric handgrip strength adjusted for body mass index.

## References

[1] Hadders-Algra M. (2018). Early human motor development: From variation to the ability to vary and adapt. Neurosci. Biobehav. Rev..

[2] Coelho Junior H., Uchida M., Gonçalves I.O., Calvani R., Rodrigues B., Picca A., Onder G., Landi F., Bernabei R., Marzetti E. (2019). Age- and Gender-Related Changes in Physical Function in Community-dwelling Brazilian adults aged 50-102 years. J. Geriatr. Phys. Ther..

[3] Marzetti E., Hwang A.-C., Tosato M., Peng L.-N., Calvani R., Picca A., Chen L.-K., Landi F. (2018). Age-related changes of skeletal muscle mass and strength among Italian and Taiwanese older people: Results from the Milan EXPO 2015 survey and the I-Lan Longitudinal Aging Study. Exp. Gerontol..

[4] Kuh D., Bassey E.J., Butterworth S., Hardy R., Wadsworth M.E.J. (2005). Grip strength, postural control, and functional leg power in a representative cohort of British men and women: Associations with physical activity, health status, and socioeconomic conditions. J. Gerontol. A Biol. Sci. Med. Sci..

[5] Rosano C., Simonsick E.M., Harris T.B., Kritchevsky S.B., Brach J., Visser M., Yaffe K., Newman A.B. (2005). Association between physical and cognitive function in healthy elderly: The health, aging and body composition study. Neuroepidemiology.

[6] Fitzpatrick A.L., Buchanan C.K., Nahin R.L., Dekosky S.T., Atkinson H.H., Carlson M.C., Williamson J.D., Ginkgo Evaluation of Memory (GEM) Study Investigators (2007). Associations of gait speed and other measures of physical function with cognition in a healthy cohort of elderly persons. J. Gerontol. A Biol. Sci. Med. Sci..

[7] Abellan van Kan G., Rolland Y., Andrieu S., Bauer J., Beauchet O., Bonnefoy M., Cesari M., Donini L.M., Gillette Guyonnet S., Inzitari M. (2009). Gait speed at usual pace as a predictor of adverse outcomes in community-dwelling older people an International Academy on Nutrition and Aging (IANA) Task Force. J. Nutr. Health Aging.

[8] Pavasini R., Guralnik J., Brown J.C., di Bari M., Cesari M., Landi F., Vaes B., Legrand D., Verghese J., Wang C. (2016). Short Physical Performance Battery and all-cause mortality: Systematic review and meta-analysis. BMC Med..

[9] Cöster M.E., Karlsson M., Ohlsson C., Mellström D., Lorentzon M., Ribom E., Rosengren B. (2018). Physical function tests predict incident falls: A prospective study of 2969 men in the Swedish Osteoporotic Fractures in Men study. Scand. J. Public Health.

[10] World Health Organization (2015). World Report on Ageing and Health. https://apps.who.int/iris/bitstream/handle/10665/186463/9789240694811_eng.pdf?sequence=1.

[11] Lorenzo-López L., Maseda A., de Labra C., Regueiro-Folgueira L., Rodríguez-Villamil J.L., Millán-Calenti J.C. (2017). Nutritional determinants of frailty in older adults: A systematic review. BMC Geriatr..

[12] Cruz-Jentoft A.J., Kiesswetter E., Drey M., Sieber C.C. (2017). Nutrition, frailty, and sarcopenia. Aging Clin. Exp. Res..

[13] Katsanos C.S., Kobayashi H., Sheffield-Moore M., Aarsland A., Wolfe R.R. (2005). Aging is associated with diminished accretion of muscle proteins after the ingestion of a small bolus of essential amino acids. Am. J. Clin. Nutr..

[14] Katsanos C.S., Kobayashi H., Sheffield-Moore M., Aarsland A., Wolfe R.R. (2006). A high proportion of leucine is required for optimal stimulation of the rate of muscle protein synthesis by essential amino acids in the elderly. Am. J. Physiol. Metab..

[15] Coelho-Júnior H.J., Milano-Teixeira L., Rodrigues B., Bacurau R., Marzetti E., Uchida M. (2018). Relative protein intake and physical function in older adults: A systematic review and meta-analysis of observational studies. Nutrients.

[16] Coelho-Júnior H.J., Rodrigues B., Uchida M., Marzetti E. (2018). Low protein intake is associated with frailty in older adults: A systematic review and meta-analysis of observational studies. Nutrients.

[17] Ten Haaf D., van Dongen E., Nuijten M., Eijsvogels T., de Groot L., Hopman M. (2018). Protein Intake and Distribution in Relation to Physical Functioning and Quality of Life in Community-Dwelling Elderly People: Acknowledging the Role of Physical Activity. Nutrients.

[18] Gregorio L., Brindisi J., Kleppinger A., Sullivan R., Mangano K.M., Bihuniak J.D., Kenny A.M., Kerstetter J.E., Insogna K.L. (2014). Adequate dietary protein is associated with better physical performance among post-menopausal women 60–90 years. J. Nutr. Health Aging.

[19] Coelho-Júnior H.J., Calvani R., Picca A., Gonçalves I.O., Landi F., Bernabei R., Cesari M., Uchida M.C., Marzetti E. (2020). Protein-related dietary parameters and frailty status in older community-dwellers across different frailty instruments. Nutrients.

[20] Volpi E., Campbell W.W., Dwyer J.T., Johnson M.A., Jensen G.L., Morley J.E., Wolfe R.R. (2013). Is the optimal level of protein intake for older adults greater than the recommended dietary allowance?. J. Gerontol. A Biol. Sci. Med. Sci..

[21] Bauer J., Biolo G., Cederholm T., Cesari M., Cruz-Jentoft A.J., Morley J.E., Phillips S., Sieber C., Stehle P., Teta D. (2013). Evidence-based recommendations for optimal dietary protein intake in older people: A position paper from the prot-age study group. J. Am. Med. Dir. Assoc..

[22] Calvani R., Miccheli A., Landi F., Bossola M., Cesari M., Leeuwenburgh C., Sieber C.C., Bernabei R., Marzetti E. (2013). Current nutritional recommendations and novel dietary strategies to manage sarcopenia. J. Frailty Aging.

[23] Ruggiero E., Di Castelnuovo A., Costanzo S., Persichillo M., Bracone F., Cerletti C., Donati M.B., de Gaetano G., Iacoviello L., Bonaccio M. (2019). Socioeconomic and psychosocial determinants of adherence to the Mediterranean diet in a general adult Italian population. Eur. J. Public Health.

[24] Mukherjea A., Underwood K.C., Stewart A.L., Ivey S.L., Kanaya A.M. (2013). Asian Indian views on diet and health in the United States: Importance of understanding cultural and social factors to address disparities. Fam. Community Health.

[25] Brown A.G.M., Houser R.F., Mattei J., Lichtenstein A.H., Folta S.C. (2019). Qualitative exploration of cultural factors influencing diet among African-, Caribbean- and US-born Blacks living in the northeast USA. J. Nutr. Sci..

[26] Juárez-Ramírez C., Théodore F.L., Villalobos A., Allen-Leigh B., Jiménez-Corona A., Nigenda G., Lewis S. (2019). The importance of the cultural dimension of food in understanding the lack of adherence to diet regimens among Mayan people with diabetes. Public Health Nutr..

[27] Instituto Brasileiro de Geografia e Estatística IBGE-Poá. https://cidades.ibge.gov.br/brasil/sp/poa/panorama.

[28] Landi F., Calvani R., Picca A., Tosato M., Martone A.M., Ortolani E., Salini S., Pafundi T., Savera G., Pantanelli C. (2018). Cardiovascular health metrics, muscle mass and function among Italian community-dwellers: The Lookup 7+ project. Eur. J. Public Health.

[29] Landi F., Calvani R., Tosato M., Martone A.M., Fusco D., Sisto A., Ortolani E., Savera G., Salini S., Marzetti E. (2017). Age-Related Variations of Muscle Mass, Strength, and Physical Performance in Community-Dwellers: Results From the Milan EXPO Survey. J. Am. Med. Dir. Assoc..

[30] Landi F., Calvani R., Picca A., Tosato M., D’Angelo E., Martone A.M., Serafini E., Ortolani E., Savera G., Salini S. (2018). Relationship between cardiovascular health metrics and physical performance in community-living people: Results from the Longevity check-up (Lookup) 7+ project. Sci. Rep..

[31] Coelho-Junior H.J., Calvani R., Gonçalves I.O., Rodrigues B., Picca A., Landi F., Bernabei R., Uchida M.C., Marzetti E. (2019). High relative consumption of vegetable protein is associated with faster walking speed in well-functioning older adults. Aging Clin. Exp. Res..

[32] Anção M., Cuppari L., Draibe A., Sigulem D. (2002). Programa de Apoio à Nutrição Nutwin: Versão 1.5.

[33] Landi F., Calvani R., Tosato M., Martone A.M., Picca A., Ortolani E., Savera G., Salini S., Ramaschi M., Bernabei R. (2017). Animal-derived protein consumption is associated with muscle mass and strength in community-dwellers: Results from the Milan Expo survey. J. Nutr. Health Aging.

[34] Guralnik J.M., Simonsick E.M., Ferrucci L., Glynn R.J., Berkman L.F., Blazer D.G., Scherr P.A., Wallace R.B. (1994). A Short Physical Performance Battery Assessing Lower Extremity Function: Association With Self-Reported Disability and Prediction of Mortality and Nursing Home Admission. J. Gerontol..

[35] Neutzling M.B., Rombaldi A.J., Azevedo M.R., Hallal P.C. (2009). Fatores associados ao consumo de frutas, legumes e verduras em adultos de uma cidade no Sul do Brasil. Cad. Saude Publica.

[36] De Oliveira Cardoso L., Sá Carvalho M., Gonçalves Cruz O., Melere C., Luft V.C., Bisi Molina M.D.C., Perim de Faria C., Benseñor I.M., Alvim Matos S.M., Mendes da Fonseca M.D.J. (2016). Eating patterns in the Brazilian Longitudinal Study of Adult Health (ELSA-Brasil): An exploratory analysis. Cad. Saude Publica.

[37] Landi F., Calvani R., Tosato M., Martone A.M., Ortolani E., Savera G., D’Angelo E., Sisto A., Marzetti E. (2016). Protein Intake and Muscle Health in Old Age: From Biological Plausibility to Clinical Evidence. Nutrients.

[38] Da Silva Gomes Ribeiro C., Corção M. (2013). The consumption of meat in Brazil: Between socio-cultural and nutritional values. Demetra.

[39] D’Alessandro A., Lampignano L., De Pergola G. (2019). Mediterranean Diet Pyramid: A Proposal for Italian People. A Systematic Review of Prospective Studies to Derive Serving Sizes. Nutrients.

[40] Pannemans D.L., Wagenmakers A.J., Westerterp K.R., Schaafsma G., Halliday D. (1998). Effect of protein source and quantity on protein metabolism in elderly women. Am. J. Clin. Nutr..

[41] McLean R.R., Mangano K.M., Hannan M.T., Kiel D.P., Sahni S. (2016). Dietary Protein Intake Is Protective Against Loss of Grip Strength Among Older Adults in the Framingham Offspring Cohort. J. Gerontol. A Biol. Sci. Med. Sci..

[42] Pallottini A.C., Sales C.H., Vieira D.A.D.S., Marchioni D.M., Fisberg R.M. (2017). Dietary BCAA Intake Is Associated with Demographic, Socioeconomic and Lifestyle Factors in Residents of São Paulo, Brazil. Nutrients.

[43] Ottestad I., Ulven S.M., Øyri L.K.L., Sandvei K.S., Gjevestad G.O., Bye A., Sheikh N.A., Biong A.S., Andersen L.F., Holven K.B. (2018). Reduced plasma concentration of branched-chain amino acids in sarcopenic older subjects: A cross-sectional study. Br. J. Nutr..

[44] Mattick J.S.A., Kamisoglu K., Ierapetritou M.G., Androulakis I.P., Berthiaume F. (2013). Branched-chain amino acid supplementation: Impact on signaling and relevance to critical illness. Wiley Interdiscip. Rev. Syst. Biol. Med..

[45] Boirie Y., Gachon P., Beaufrère B. (1997). Splanchnic and whole-body leucine kinetics in young and elderly men. Am. J. Clin. Nutr..

[46] Wall B.T., Gorissen S.H., Pennings B., Koopman R., Groen B.B.L., Verdijk L.B., Van Loon L.J.C. (2015). Aging is accompanied by a blunted muscle protein synthetic response to protein ingestion. PLoS ONE.

[47] Mao D., Chen F., Wang R., Bai P., Zhang Y., Zhao W., Chen J., Yang L., Yang X., Li M. (2020). Protein Requirements of Elderly Chinese Adults Are Higher than Current Recommendations. J. Nutr..

[48] Rafii M., Chapman K., Elango R., Campbell W.W., Ball R.O., Pencharz P.B., Courtney-Martin G. (2015). Dietary Protein Requirement of Men >65 Years Old Determined by the Indicator Amino Acid Oxidation Technique Is Higher than the Current Estimated Average Requirement. J. Nutr..

[49] De Marchi R.J., Hugo F.N., Hilgert J.B., Padilha D.M.P. (2008). Association between oral health status and nutritional status in south Brazilian independent-living older people. Nutrition.

[50] McGrath R., Stastny S., Casperson S., Jahns L., Roemmich J., Hackney K.J. (2019). Daily Protein Intake and Distribution of Daily Protein Consumed Decreases Odds for Functional Disability in Older Americans. J. Aging Health.

[51] Shim J.-S., Oh K., Kim H.C. (2014). Dietary assessment methods in epidemiologic studies. Epidemiol. Health.

